# A high-performance Ni-CeO_2_/Ni/Ni-Y_2_O_3_·ZrO_2_ three-layer anode for direct iso-octane feeding of solid oxide fuel cells

**DOI:** 10.1098/rsos.220227

**Published:** 2022-07-20

**Authors:** Kazuya Sasaki, Ikuma Takahashi, Kodai Kuramoto, Kiyoto Shin-mura

**Affiliations:** ^1^ Graduate School of Science and Technology, Hirosaki University, 3 Bunkyo-cho, Hirosaki, Aomori, 036-8561, Japan; ^2^ Faculty of Engineering, Department of Advanced Materials Science and Engineering, Chiba Institute of Technology, 2-17-1 Tsudanuma, Narashino, Chiba 275-0016, Japan

**Keywords:** solid oxide fuel cell (SOFC), anode catalyst, carbon deposition, iso-octane fuel, steam reforming

## Abstract

Solid oxide fuel cells (SOFCs) directly fed with iso-octane are expected to be power sources for mobile devices and automobiles. However, the conventional anode catalysts nickel (Ni) or cerium oxide (CeO_2_) used for direct feeding of iso-octane do not suppress carbon deposition or generate high power. In this study, we investigated the Ni-CeO_2_/Ni/Ni-yttria-stabilized-zirconia (YSZ) three-layer anode to establish the suppression of carbon deposition and high-power generation in the SOFC. The anode consists of a Ni-CeO_2_ catalyst layer as the top layer, an Ni catalyst layer as the second layer, and a Ni-YSZ catalyst layer as the third layer on top of the electrolyte. The concept of the three-layer anode is as follows: fuel reforming occurs in the Ni-CeO_2_ layer, the reformed H_2_ or CO is electrochemically oxidized in the Ni-YSZ catalyst layer, and the Ni catalyst middle layer prevents the reaction between YSZ and CeO_2_. Scanning electron microscopy and electrochemical characterization confirmed carbon deposition suppression and improved power generation. The anode showed no carbon deposition and generated high-power, 600 mA cm^−2^ and 150 mW cm^−2^, at 950°C and a steam/carbon ratio of 3.0. Additionally, we discuss the fuel reforming reactions on the three-layer electrode by the results of exhaust gas analysis.

## 1. Introduction

Fuel cells are highly efficient power generation systems that directly convert fuel energy into electric power without the restriction of the Carnot efficiency. Additionally, fuel cells are expected to be used as portable smaller power generation devices because they have few scale disadvantages. Notably, solid oxide fuel cells (SOFCs) are power generation systems that can use high-energy hydrocarbons as fuel, and therefore, are expected to be employed as energy conversion devices to provide power sources in robots, households, or hybrid systems with internal combustion. 2,2,4-Trimethylpentane (iso-octane, C_8_H_18_) is easy to handle because it is a liquid at ambient temperature and easily vapourizes and is a major component of gasoline. For this reason, SOFCs that directly supply iso-octane as a fuel have recently been investigated as power sources for mobile devices and vehicles [1–25]. The SOFCs that directly supply iso-octane are generally operated with a steam reforming method, which extracts reactive species of hydrogen (H_2_) and carbon monoxide (CO) from the fuel during power generation. A nickel (Ni) catalyst is commonly used as an anode catalyst for steam reforming owing to its high electric conductivity, high thermal stability and high reactivity to H_2_ oxidation. However, carbon deposition occurs with an Ni catalyst when using a high molecular hydrocarbon as fuel at a low steam/carbon (S/C) ratio [1,3,5–7,17,24–40]. Based on the previous study, the carbon deposition is caused by a short supply of oxygen species on the Ni catalyst when it oxidizes the lower hydrocarbons, such as methane (CH_4_) produced by the cracking reaction of the higher hydrocarbons fed as fuel [37,41,42]. To counter this phenomenon, Gorte *et al*. reported that the copper (Cu)-cerium oxide (CeO_2_) anode suppresses carbon deposition by supplying oxygen species from the reduction of CeO_2_ [31,43–45]. Furthermore, Marnellos *et al*. demonstrated that the use of Cu-CeO_2_ as an anode catalyst improves the power generation performance of the SOFCs directly fed iso-octane as fuel [9,16,24,25]. However, compared to SOFCs feeding H_2_ or low hydrocarbons as fuels, power generation is still lower and carbon deposition is not completely suppressed. In addition, CeO_2_ is easy to react with the yttria-stabilized zirconia (YSZ) of the conventional SOFC electrolyte at high temperatures, which results in low power generation owing to the formation of cerium-zirconate that has low oxide ion conductivity [46]. Thus, the compatibility of the suppression of carbon deposition and the generation of high power has not been established on the SOFC anode directly fed iso-octane as fuel.

In this study, we investigated a new structural design of the three-layer anode, Ni-CeO_2_/Ni/Ni-YSZ, to determine the compatibility of the suppression of carbon deposition and the generation of high power in the SOFC directly fed iso-octane. The design of the three-layer anode has a Ni-CeO_2_ catalyst layer as the top layer, a Ni catalyst layer as the second layer, and a Ni-YSZ catalyst layer as the third layer on top of the YSZ electrolyte. The concept of the three-layer structural function is shown as follows. Fuel reforming effectively occurs in the Ni-CeO_2_ top layer because Ni catalyses steam reforming and CeO_2_ catalyses the suppression of carbon deposition. Subsequently, the extracted H_2_ or CO from iso-octane is electrochemically oxidized in the third Ni-YSZ catalyst layer. The function of the Ni catalyst as the second layer was to prevent the reaction between YSZ and CeO_2_. In this paper, the performance of the three-layer anode of Ni-CeO_2_/Ni/Ni-YSZ is confirmed based on the observation of carbon deposition and power generation properties of SOFCs directly fed iso-octane as fuel. Additionally, the reforming reactions on the three-layer anode are discussed by the results of exhaust gas analysis.

## 2. Experimental

### 2.1. Sample preparation

**Figure 1.**
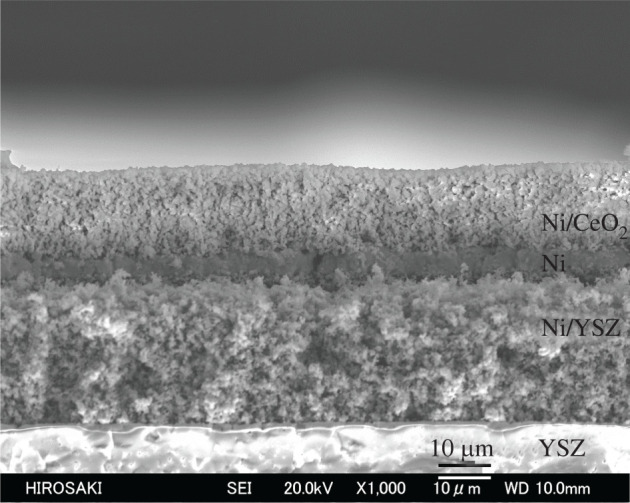
Scanning electron microscopy image of the prepared Ni-CeO_2_/Ni/Ni-YSZ three-layer anode.

The electrolyte support used was 8 mol% yttrium oxide (Y_2_O_3_)-stabilized zirconium dioxide (ZrO_2_) powder (8YSZ; Daiichi Kigenso Kagaku Kogyo, Osaka, Japan); it was formed by press moulding, and then sintered at 1400°C for 6 h in air. The diameter and thickness of the electrolyte support was 18 mm and 1.0 mm, respectively. The cathode electrode was prepared using platinum (Pt) paste (TR-7907, lot no. L3000186015023; Tanaka Kikinzoku Kogyo, Tokyo, Japan), in which only high purity Pt powder was dispersed in the organic binder. The Pt paste was applied and sintered at 1300°C for 1 h in air. The cathode electrode was coated on the prepared electrolyte pellet in a square 0.5 mm grid with 8 mm on one side. The reference electrode with a width of 0.5 mm was coated around the centre of the side surface of the electrolyte pellet with a thickness of 5 μm. The prepared Pt electrodes were dense and in the same shape, and the triple-phase boundary length as a reaction field was the same for all samples; therefore, the influence of the difference in the cathode characteristics of each sample was removed. The Ni-CeO_2_/Ni/Ni-YSZ three-layer anode was prepared as follows. Nickel oxide (NiO) powder was obtained by a thermal decomposition from nickel carbonate (NiCO_3_) powder (Kanto Kagaku, Tokyo, Japan) at 600°C for 5 h. The NiO and 8YSZ powders with a mass ratio of 69 : 31 were mixed in water (H_2_O) using a planetary ball mill. The sample of 1 g and pure water of 100 g were poured into a zirconia pot of 250 ml and a zirconia ball was added with a diameter of 5 mm of 400 g. Grinding and mixing condition was the orbital speed of 200 rpm for 24 h. After a planetary ball mill, the slurry was dried. The dry mixed powder of NiO-YSZ for the Ni-YSZ third layer was obtained by pounding in a mortar. Here, a mass ratio of 69 : 31 for NiO and 8YSZ powders corresponds to a volume ratio of 50 : 50 for Ni and 8YSZ under working anode condition, which can form the good three-phase boundary. For the preparation of the NiO-CeO_2_ mixed powder for the first Ni-CeO_2_ layer, the NiO and CeO_2_ powders (Daiichi Kigenso Kogyo) were mixed at a mass ratio of 55 : 45, corresponding to a volume ratio of 50 : 50 for Ni and CeO_2_, and the subsequent procedure was the same as the preparation of the Ni-YSZ cermet. The mixed NiO-YSZ, NiO-CeO_2_, and NiO powders were dispersed in an appropriate amount of solvent (α-terpineol) containing 30 mass% of ethyl cellulose and 0.5 mass% of surfactant (Duomin TDO, Lion, Tokyo, Japan) by powder weight, resulting in NiO-YSZ, NiO-CeO_2_ and NiO pastes. The NiO-YSZ paste was applied to the centre of the opposite face of the 8YSZ electrolyte pellet in a circle, with a diameter of 8 mm by screen printing. The obtained cell was dried at 110°C for 30 min and then sintered at 1300°C for 10 h in air. Subsequently, the NiO paste was coated on the NiO-YSZ electrode, and then the NiO-CeO_2_ paste was coated on the dried NiO paste. The cell with the three-layer anode was sintered at 1300°C for 10 h in air, and finally, reduced at 950°C for 15 h in a H_2_ atmosphere. The prepared three-layer anode of Ni-CeO_2_/Ni/Ni-YSZ is shown in figure 1.

### 2.2. Evaluation of power generation properties

The SOFC test was evaluated using a single cell. The open-circuit potential (OCP), and the anode overpotential during power generation were investigated using an electrochemical workstation (SP150, Biologic SAS, France). The anode overpotential of the Ni-CeO_2_/Ni/Ni-YSZ three-layered anode as a working electrode during the power generation was measured by linear sweep voltammetry with a three-electrode system. After setting the cell, the temperature was raised to 950°C at 100°C h^–1^ in nitrogen (N_2_). Then, high-purity oxygen (O_2_) was flowed onto the cathode and reference electrode sides, and humidified hydrogen (3% H_2_O) was flowed onto the anode side. After several hours, the temperature dependence of the OCP measurement was carried out at 950°C to lower temperatures 900, 850, 800, 750 and 700°C at a rate of –5°C h^−1^.

The S/C dependence of the OCP measurement was conducted as below: iso-octane and steam at equilibrium vapour pressure were supplied to the single cell by bubbling a carrier gas (helium (He) or argon (Ar)) through iso-octane or water. The octane bubbler had a temperature of 30°C and gas flow rates of 10–14 ml min^−1^. The carrier gas flow rate and the temperature of the water bubbler were changed in ranges of 75–98 ml min^−1^ and 50–70°C, respectively. Then, the S/C ratio was varied from 5.0 to 1.0. The octane and steam inlet concentrations (*p*_H_2__O__/*p*_octane_) excluding the balance gas (He) were 28.82/0.72, 21.46/0.67, 20.87/0.86, 14.47/0.91 and 9.19/1.16 (%/%) when S/C = 5.0, 4.0, 3.0, 2.0 and 1.0, respectively.

The presence of carbon deposition was confirmed by analysis of the electrode surface using scanning electron microscopy (SEM) (JEOL, JSM-7000F, Tokyo, Japan), and the formation of the reaction phases of YSZ and CeO_2_ was analysed by energy-dispersive X-ray spectrometry (EDS) (JEOL, JED-2300F, Tokyo, Japan).

### 2.3. Exhaust gas analysis

The exhaust gas composition from the cells in the open-circuit state was analysed by gas chromatography (GC; GC-3200, GL Sciences) with thermal conductivity detection. As the carrier gas, Ar and He were used for the analysis of H_2_ and the other gases in the exhaust gas, respectively. Quantification of the results was performed using a calibration curve. The inorganic components of the gas and methane were detected using an active carbon column at 50°C, while the organic components were detected using a Porapak Q column at 150°C. Measurements from the two different columns were calibrated using the measured values of the same concentration of methane. The peaks derived from steam were removed from the exhaust gas of the cell using a cold trap because steam had a broad GC peak with strong intensity. The amounts of remaining H_2_O were estimated based on the theoretical mass balance.

## 3. Results and discussion

### 3.1. Temperature dependence of open-circuit potential with a steam/carbon ratio of 1.0

**Figure 2.**
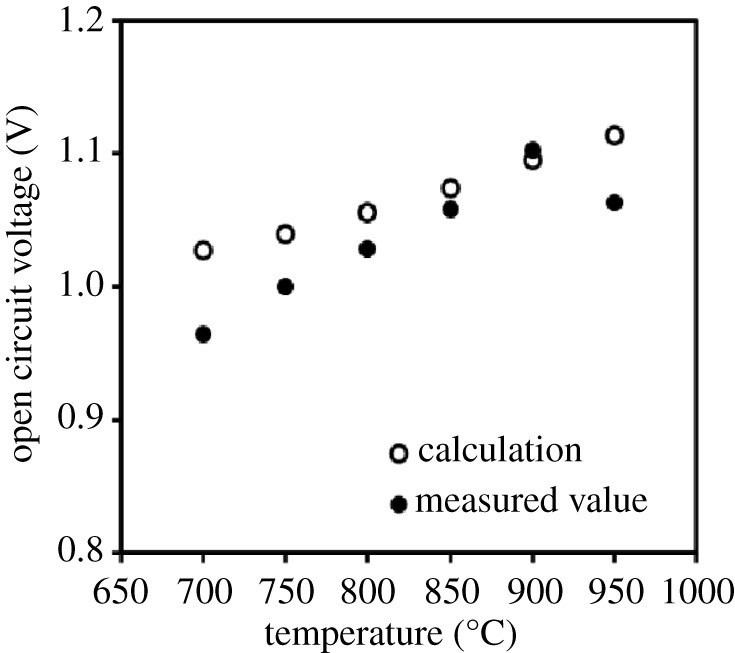
Open-circuit potentials of a single cell operated with internal steam reforming of iso-octane at a steam/carbon (S/C) ratio of 1.0 at various cell temperatures. Closed circles, measured values; open circles, calculated values estimated using the Nernst equation based on the equilibrium oxygen partial pressure calculated by the thermodynamic database MALT when iso-octane and water were supplied at an S/C = 1.0 and 700–950°C.

The OCP at various temperatures with an S/C of 1.0 is shown in figure 2. The plot shows the mode value, and the error bar indicates minimum and maximum values for the 1 h measurement in this figure. In a fuel cell using an oxygen ion conductor as the electrolyte, the OCP was determined as the function of the temperature and the oxygen activity on both sides of the electrolyte. In the figure, the theoretical OCP values calculated using the Nernst equation when iso-octane and H_2_O are supplied at an S/C = 1.0 and at the temperature range of 700–950 °C are also shown. For the calculation, the equilibrium oxygen partial pressure (*p*_O2_) was estimated by the thermodynamic database Materials Oriented Little Thermodynamic Database (MALT). The measurement values did not exactly match the calculated values because the measured values are decided by the experimental state such as the actual temperature of cell and *p*_O2_. However, the same temperature dependence was exhibited in the temperature range below 900°C. The gaps between 700 and 850°C is considered to originate from the temperature of flow gas which could be lower than that of set, since the temperature uniformity zone in the tubular furnace is generally narrow at lower temperature. By contrast, a rise in temperature from 900°C to 950°C reduced the OCP, contrary to the predicted value. This decrease of the OCP from 900 to 950°C may be owing to the impact of increasing *p*_O2_ by the oxygen generated from CeO_2_. The operation temperatures of 900 and 950°C are considered to be suitable because the high OCP provided high power generation. Accordingly, the operation temperature for SOFC using the three-layer anode was employed at 950°C in this study.

**Figure 3.**
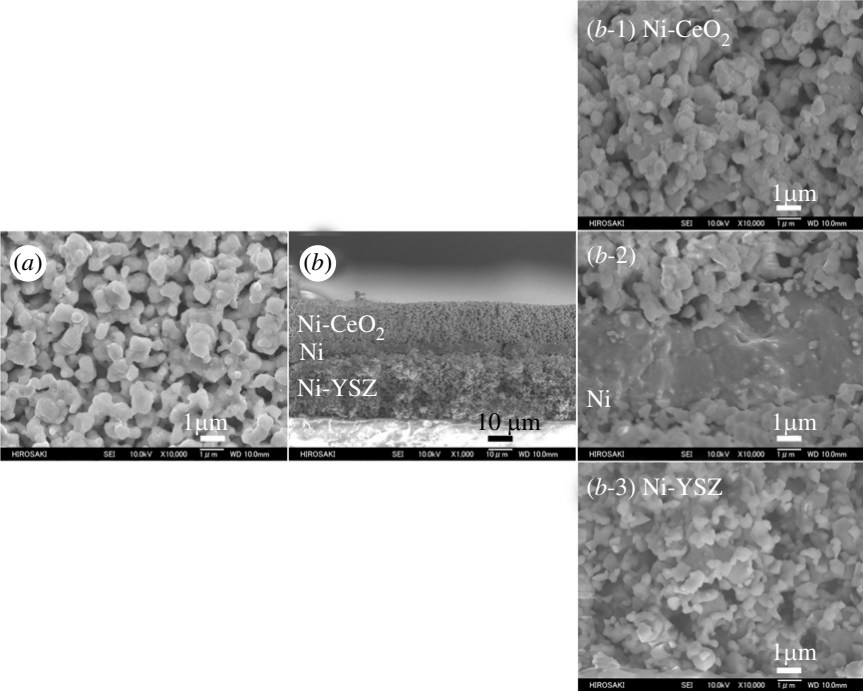
Scanning electron microscopy images of the Ni-CeO_2_/Ni/Ni-YSZ three-layer anode after operation at 950–700°C with a steam/carbon ratio of 1.0: (*a*) surface and (*b*) fractured cross section; (*b*-1) Ni-CeO_2_ layer, (*b*-2) Ni layer, (*b*-3) Ni-YSZ layer. The temperature just before the image was taken was 700°C.

Subsequently, SEM images of the surface and cross section of the Ni-CeO_2_/Ni/Ni-YSZ three-layer anode after OCP operation from 950 to 700°C with an S/C ratio of 1.0 are shown in figure 3. Although OCP operation is the difficult condition to suppress the carbon deposition owing to the shortage of oxygen supply through the electrolyte, there was no carbon deposition on the three-layer electrode. Thus, the three-layer electrode in OCP operation was confirmed to provide the internal steam reforming without carbon deposition between 700 and 950°C with an S/C ratio of 1.0.

### 3.2. Steam/carbon dependence of open-circuit potential at 950°C

**Figure 4.**
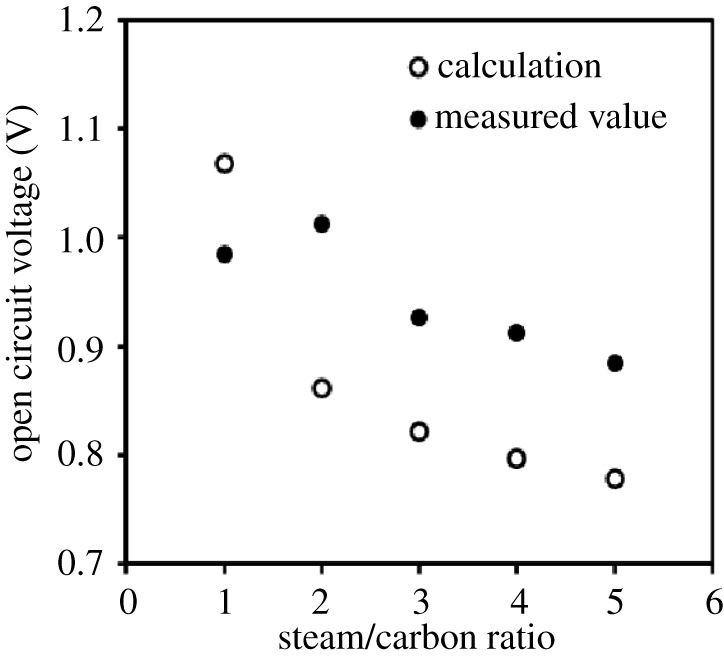
Open-circuit potentials of a single cell operated with internal steam reforming of iso-octane at 950°C under various steam/carbon (S/C) ratios of 1.0 to 5.0. Closed circles, measured values; open circles, calculated values estimated using the Nernst equation based on the equilibrium oxygen partial pressure calculated by the thermodynamic database MALT when iso-octane and water were supplied at an S/C = 1.0–5.0 and 950°C.

**Figure 5.**
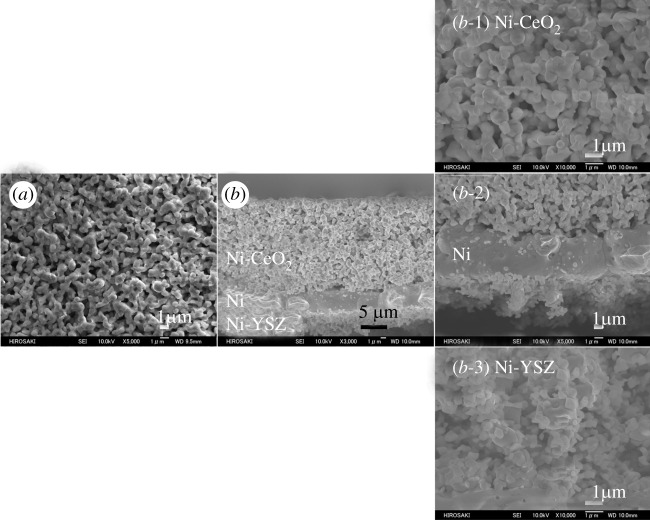
Scanning electron microscopy images of the Ni-CeO_2_/Ni/Ni-YSZ three-layer anode after operation at 950°C with steam/carbon (S/C) ratios of 5.0 to 1.0: (*a*) surface and (*b*) fractured cross section; (*b*-1) Ni-CeO_2_ layer, (*b*-2) Ni layer, (*b*-3) Ni-YSZ layer. The S/C ratio just before the image was taken was 1.0.

The OCP measured at various S/C ratios and 950°C is shown in figure 4. In the figure, the OCP values estimated using the Nernst equation based on the equilibrium *p*_O2_ when iso-octane and H_2_O were supplied at an S/C = 1.0–5.0 and 950°C are also shown. The measured values did not correspond the calculated values either in this experiment. However, the same OCP trend between the measured value and calculated value, decreasing OCP value with increasing S/C ratio, is obtained above except at S/C = 1.0. As described above regarding the temperature dependence of the OCP, although the actual temperature of cell and *p*_O2_ impact the measured OCP, the actual temperature of the cell can be ignored because 950°C is enough to uniformize the temperature of the tubular furnace in this experiment. This decrease of the OCP would be attributed to the decrease of *p*_O2_ with an increasing S/C ratio. Namely, the supply of H_2_O may feed lower than that of set because the increase of flow rate was needed above S/C = 2.0. Regarding S/C = 1.0, the calculated OCP value could be higher than measured OCP owing to an increase of *p*_O2_ by the oxygen generated from CeO_2_ as above. The OCP of an S/C = 3.0 was modestly high and stable, therefore, S/C = 3.0 was employed as the condition of power generation evaluation at 950 °C. Subsequently, SEM images after the S/C dependence evaluation are shown in figure 5. There was also no carbon deposition under any evaluated S/C ratios in the three-layer anode.

### 3.3. Power generation properties

**Figure 6.**
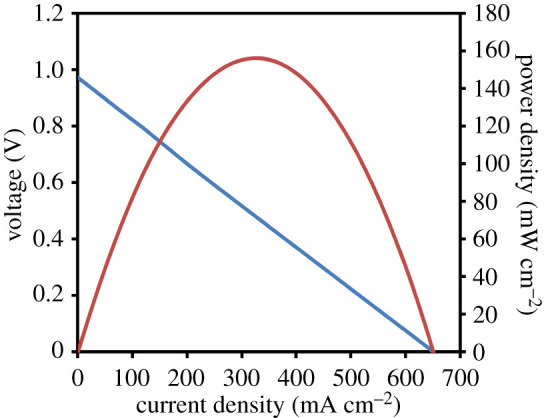
Current density-voltage (*J-V*) and current density-power (*J-P*) curves of a single cell operated with internal steam reforming of iso-octane at a steam/carbon ratio of 3.0 at 950°C.

**Figure 7.**
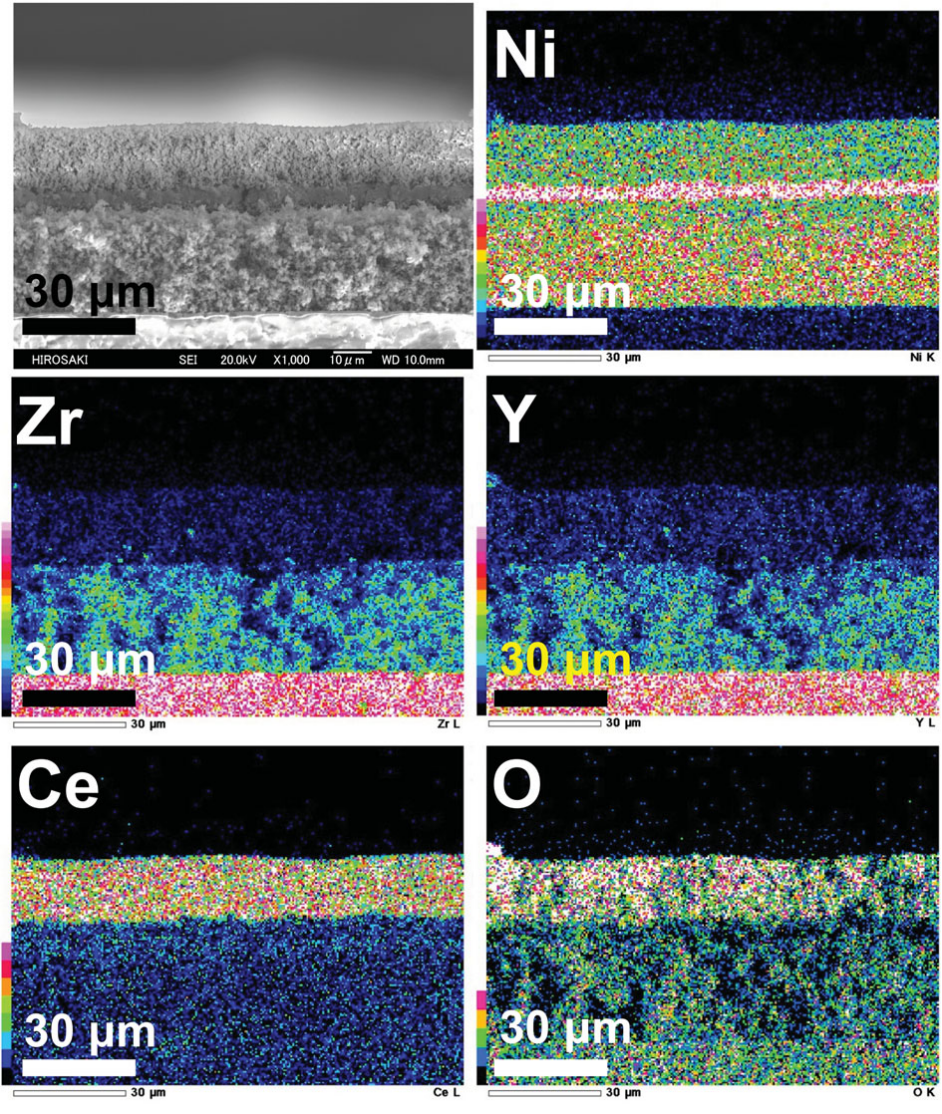
Energy-dispersive X-ray spectrometry images of the Ni-CeO_2_/Ni/Ni-YSZ three-layer anode after the measurement of power generation properties at 950°C with steam/carbon ratios from 5.0 to 1.0.

The evaluation of power generation properties using the three-layer anode at the optimal condition of 950°C with an S/C = 3.0 is shown in figure 6. The power generation using the three-layer anode is as good as that of other excellent SOFCs directly fed iso-octane [5,7,9–11,13,15,16,18,19,25]. In addition, figure 7 shows EDS images of the three-layer anode of Ni-CeO_2_/Ni/Ni-YSZ after the evaluation of the power generation properties. The reactive layer between CeO_2_ and YSZ, a low conductivity material layer, was not observed owing to the insertion of the Ni second layer. Namely, the formation of the reactive layer was suppressed by the insertion of the Ni second layer. One of the possibilities of high-power generation is attributed to be suppression of the formation of the reactive layer, which indicated that the three-layer anode worked as well as the concept. The functional concept of the first and third layer will be discussed based on the fuel reforming reaction at the later part.

### 3.4. Fuel reforming reactions on the three-layer anode

**Figure 8.**
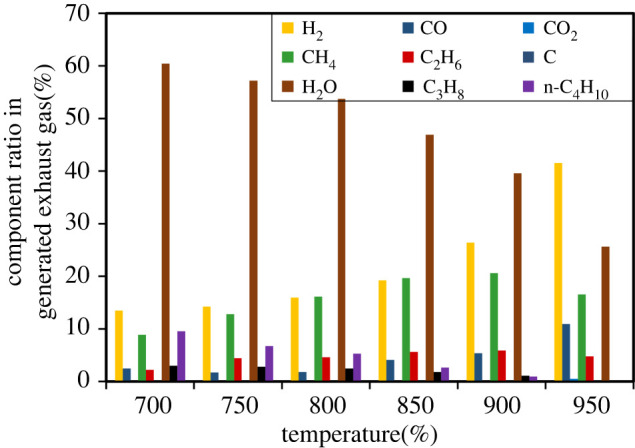
Outlet gas composition during internal steam reforming of iso-octane on a Ni-CeO_2_/Ni/Ni-YSZ three-layer laminated anode under open-circuit conditions between 700°C and 950°C with a steam/carbon ratio of 1.0.

The gas exhaust analysis by internal steam reforming was conducted at the OCP and S/C = 1.0 because the condition of OCP operation and low S/C is most likely to have occurred because of the carbon deposition owing to the shortage of oxygen supply in the experimental conditions. The temperature dependence of the gas composition is shown in figure 8. The vertical axis is the component ratio in the generated exhaust gas (not including the carrier gas). By increasing the temperature between 700 and 900°C, the product ratio of H_2_, CO, CH_4_ and ethane (C_2_H_6_) increased, and that of H_2_O, propane (C_3_H_8_), and n-butane (C_4_H_10_) decreased. At 950°C, the product ratio of H_2_ and CO dramatically increased and that of CH_4_ and C_2_H_6_ decreased. In the entire range of measured temperatures, unreformed iso-octane and any hydrocarbons (C > 5) in the exhaust gas were not detected. Here, we infer some of the possible iso-octane reforming reactions and verify the validity of the concept of the three-layer anode based on the exhaust gas composition under the OCP. According to the literature, Ni catalyses the cracking reaction, which breaks a methyl group from hydrocarbons [37,41,42]. Additionally, Sasaki *et al*. suggested that H_2_ and CO are produced by the hydrogenation of the methyl radical (CH_3_•) and the ethyl radical (C_2_H_5_•) in the reforming process of n-octane by the Ni catalyst [47]. Based on the papers, the terminal methyl and ethyl groups of iso-octane would be cleaved, and transformed to CH_3_• and C_2_H_5_•, shortening carbon chain length. Similarly, the remaining lower hydrocarbons were presumed to become a shorter carbon chain in the same reaction. Thus, it was estimated that the next sequential reaction had probably occurred on the anode in this study. Initially, the carbon number of hydrocarbon chains decreased to 3 or 4 (reaction (3.1)):

3.1 $$\mathrm{iso}-C_{8}H_{18}\to x{\mathrm{CH}}_{3}∙+yC_{2}H_{5}∙+z\;\mathrm{remaining}\;\mathrm{hydrocarbon}\;\mathrm{radical}\;(C3\text{–}C4).$$

x+2y+(C3–C4)z=8

The value of *x* and *y* increased and the value of *z* decreased at higher temperatures, resulting in higher production of CH_4_ and C_2_H_6_ and lower production of C_3_H_6_ and n-C_4_H_10_. Subsequently, the highly active CH_3_• and C_2_H_5_• transformed to CH_4_ and C_2_H_6_ by adding the hydrogen radical (H•) formed from H_2_O (reactions (3.2) and (3.3)):

3.2 $${\mathrm{CH}}_{3}∙+H_{2}O\to {\mathrm{CH}}_{4}+\mathrm{OH}∙$$

3.3 $$C_{2}H_{5}∙+H_{2}O\to C_{2}H_{6}+\mathrm{OH}∙$$

Furthermore, as demonstrated by thermodynamics and a review of previous studies [48], the formed CH_4_ and C_2_H_6_ probably changed to H_2_ and CO by steam reforming reactions (reactions (3.4) and (3.5):

3.4 $${\mathrm{CH}}_{4}+H_{2}O\to 3H_{2}+\mathrm{CO}$$

3.5 $$C_{2}H_{6}+2H_{2}O\to 5H_{2}+2\mathrm{CO}$$

As reported by Huang *et al*. [48], the carbon deposition happens on the ordinal Ni anode because the supply of oxygen radicals originating from a hydroxyl radical by reactions (3.2) and (3.3) is slow, and the oxygen separated from the constituent oxide materials of the anode plays an important role to suppress carbon deposition. In this study, no carbon deposition was observed. Thus, similar to previous studies [49–51], oxygen radicals must have been generated by the reduction of CeO_2_ on the top layer of Ni-CeO_2_ and oxidized carbon to remove it. Besides, the decrease in CH_4_ and C_2_H_6_ and increase in H_2_ and CO would be owing to the difference in the reaction rate at 900 and 950°C. In other words, the kinetics of the steam reforming reactions from CH_4_ and C_2_H_6_ to H_2_ and CO were faster than the CH_4_ and C_2_H_6_ generation reactions at temperatures above 900°C.

Taken together, the iso-octane reforming reactions on the three-layer anode are suggested as follows: iso-octane was reformed to CH_4_ and C_2_H_6_ by cracking and hydrogenation, subsequently, the produced CH_4_ and C_2_H_6_ would change to H_2_ and CO via steam reforming reactions on the Ni catalyst in the top layer. Carbon was not deposited because carbon produced by the disproportional reaction would convert to CO by supplied O• from the reduction of CeO_2_. Namely, the concept of the three-layer anode would work well.

## 4. Conclusion

We investigated the Ni-CeO_2_/Ni/Ni-YSZ three-layer anode to show the compatibility of the suppression of carbon deposition and the improvement of power generation for SOFC directly fed iso-octane. As a result, the suppression of carbon deposition at the OCP conditions of 700–950°C with an S/C ratio from 1.0–5.0 was confirmed, and the power generation was 600 mA cm^−2^ and 150 mW cm^−2^ at 950°C with an S/C of 3.0. Consequently, the compatibility of a three-layer anode was verified. For the next step, we are investigating the long-term stability of the three-layer anode. The multilayer electrode concept is expected to improve not only various properties but also add new functions and develop new technology.

## Data Availability

Each data of figures 2, 4, 6 and 8 is shown in the tab of 'Figure Data' as an Excel file. Columns are labelled properly. The 'Figure Data' file does not have any 'new' datasets other than those used to produce the actual figures. Our datasets are deposited at Dryad: https://doi.org/10.5061/dryad.k98sf7m7j [52].
